# Editorial: The earliest-born cortical neurons as multi-tasking pioneers: expanding roles for subplate neurons in cerebral cortex organization and function, volume II

**DOI:** 10.3389/fnana.2023.1211678

**Published:** 2023-05-17

**Authors:** Yuichiro Oka, Makoto Sato, Shen-Ju Chou

**Affiliations:** ^1^Division of Developmental Neuroscience, United Graduate School of Child Development (UGSCD), Osaka University, Suita, Japan; ^2^Department of Anatomy and Neuroscience, Graduate School of Medicine, Osaka University, Suita, Japan; ^3^Institute of Cellular and Organismic Biology, Academia Sinica, Taipei, Taiwan

**Keywords:** cerebral cortex, subplate, layer 6b, interstitial white matter neurons, human cortical development, disease model

The subplate is a versatile layer in the developing neocortex of the mammalian brain. It forms a distinct layer beneath the developing cortical plate, serving as an interface between the cortical plate and the intermediate zone that contains growing axons and migrating neurons. Subplate neurons are transient recipients of thalamocortical inputs with functional synapses until the thalamocortical axons reach layer IV where they establish persistent connections. In addition, subplate neurons form temporal synapses with later-born neurons migrating to the cortical plate. Other features of the subplate are its heterogeneity and its massive loss during the postnatal development. Subplate impairment is implicated in mental/neurodevelopmental disorders.

After the publication of the initial volume of the Research Topic (Sato and Chou, 2020), the research field of cortical subplate has exhibited significant expansion with more focus on subplate in human cortex. In the fetal cortex, during mid-gestation, the subplate becomes elaborated with three sublayers and more than twice as thick as the cortical plate above it (Kostović et al., 2019). Molecular (Miškić et al., 2021; Smith et al., 2021; Žunić Išasegi et al., 2022; Junaković et al., 2023; Kopić et al., 2023) and *in utero* functional MRI studies described temporal changes of cellular components and morphological dynamics of the three-layered subplate (Vasung et al., 2021; Taymourtash et al., 2023; Wilson et al., 2023). Another focus in the research field is implication of the subplate in disease conditions. For example, 45% increase of NeuN-positive neurons was reported in the subplate of ASD subjects (Avino and Hutsler, 2021).

Studies in rodents have deepened the understanding of molecular mechanisms that underlie cortical circuit formation during development. Subplate-specific knockout of *Arid1a*, a chromatin remodeler gene, exhibited severe disruption of both subplate organization and co-fasciculation of axons from the subplate and thalamus (Doyle et al., 2021). Transcription factor LHX2 expression in neuronal progenitors of subplate neurons at embryonic day 11.5 is indispensable for correct penetration and pathfinding by thalamocortical axons in the cortical plate (Pal et al., 2021). Peripheral sensory stimuli during early postnatal development affect local circuit formation that involves both cortical plate and subplate (Mehra et al., 2022; Mukherjee et al., 2022; Xue et al., 2022).

In the postnatal cortex, layer VIb contains surviving subplate remnant cells. Circuitry and sensory responses of these neurons were also reported. Among the heterogeneous population of layer VIb neurons in the barrel cortex, fusiform neurons have local axons within the subplate while pyramidal neurons have axons extending throughout the cortical layers (Ghezzi et al., 2021). In the visual cortex of juvenile mice, layer VIb neurons showed broader tunings for visual parameters compared with layer 2/3 neurons and layer 6a neurons as well as the ocular dominance plasticity after monocular deprivation during critical period (Yoneda et al., 2023).

This Research Topic “The Earliest-Born Cortical Neurons as Multi-Tasking Pioneers: Expanding Roles for Subplate Neurons in Cerebral Cortex Organization and Function, Volume II” consists of four Original Research articles reporting new findings on molecular, cellular and circuitry aspects of subplate, including those with anatomical study of subplate in human fetus and mouse model of schizophrenia.

Alzu'bi and Clowry reported expression of Annexin V (ANXA5) in the upper subplate of human fetal cortex at mid-gestational stage (PCW19). Although its functional relevance in the upper subplate awaits further investigation, ANXA5 may be involved in sorting/guidance of thalamocortical axons as these axons spend some time (“waiting” period) in the upper subplate before entering the cortical plate.

Gellért et al. demonstrated in mice that at the day of birth, subplate neurons in the primary somatosensory cortex (S1) and the primary motor cortex (M1) formed functional connections, preceding the connectivity between S1 and the secondary somatosensory cortex and two days earlier than previously reported anatomical projection from S1 to M1 (Tiong et al., 2019). Their functional connectivity analysis suggested that information flows from the subplate to the cortical layers in early postnatal cortex, emphasizing the relevance of the subplate as a hub for cortical network formation.

Tsai et al. studied white matter neurons and layer VIb neurons, both of which are the remnant populations of the subplate neurons, in *Disc1* heterozygous mouse. Increased number of NeuN-positive white matter neurons and CTGF-positive layer VIb neurons, and reduced number of Cplx3-positive layer VIb neurons were observed in this mouse model of schizophrenia. In addition, horizontal neurons showed abnormal dendritic morphology in the mutant. Mis-organization of neuronal subtypes and circuits in the subplate may be related to the altered brain functions in the *Disc1* heterozygous mutant that show schizophrenia-like behaviors.

Kement et al. examined the expression of neuroserpin, a serine protease inhibitor, in the somatosensory cortex of developing and adult mice, and its role in cortical lamination and synaptic proteome. The expression was detected in the bottom of Tbr1-positive deep layers, corresponding to subplate/layer VIb, which is consistent with a previous report (Kondo et al., 2015). The formation of subplate as well as that of other cortical layers seems to be normal in the absence of neuroserpin.

In conclusion, the four articles provided new information on neuroanatomy of the subplate and its cell-type dependent alterations in a schizophrenia model. These findings in conjunction with the studies outside this Research Topic should be clues to uncover the logics of construction and regulation of heterogeneous components of the subplate, eventually leading to holistic understanding of the neuronal circuits and functions of the subplate in the developing and mature cerebral cortex.

Finally, we would like to thank all the contributors and hope this Research Topic helps facilitate future researches to reveal the underlying mechanisms of subplate development, functions, evolution, and pathophysiology in brain disorders.

## Author contributions

All authors listed have made a substantial, direct, and intellectual contribution to the work and approved it for publication.
